# 3-D micro-architecture and mechanical response of soil cemented via microbial-induced calcite precipitation

**DOI:** 10.1038/s41598-018-19895-w

**Published:** 2018-01-23

**Authors:** Dimitrios Terzis, Lyesse Laloui

**Affiliations:** Laboratory for Soil Mechanics, Swiss Federal Institute of Technology, Lausanne, Switzerland

## Abstract

We introduce the application of microbial-induced calcite precipitation via the ureolytic soil bacterium Sporosarcina Pasteurii in freeze-dried form, as a means of enhancing overall MICP efficiency and reproducibility for geotechnical engineering applications. We show that the execution of urea hydrolysis and CaCO_3_ precipitation persist as a “cell-free” mechanism upon the complete breakdown of rehydrated cell clusters. Further, strength and stiffness parameters of bio-cemented sands are determined. Medium-grained bio-cemented sand yields compressive strengths up to 12 MPa while, surprisingly, fine-grained sand yields up to 2.5 MPa for similar bond contents. To understand the observed discrepancies, we undertake a systematic study of the bio-cemented material’s microstructure, by combining a series of microstructural inspection tools. The study extends beyond conventional qualitative and textural characterization and provides with new insight into the material’s peculiar 3D micro-architecture. We apply a new methodology towards quantifying crucial microscopic characteristics such as the particle sizes of the crystalline bond lattice, the bond-grain contacts and particle orientations. Bonds are found to exhibit distinctive geometries and morphologies when MICP applies to different base materials. We thus contribute to the debate on the importance of factors affecting: (i) MICP efficiency, (ii) the mechanical response and (iii) peculiar micro-architecture of bio-improved geo-materials.

## Introduction

The quest for novel, performant materials and solutions in engineering problems is driven nowadays by coupling technical innovation with economic efficiency and positive environmental impact. This quest is more current and pertinent than ever in the construction industry. Especially in urban areas “good” soils are already taken and the remaining land often requires extensive soil stabilization and strengthening works to secure the integrity of engineering projects and mitigate long-term risks related to failures and environmental threats.

This study puts the focus on bio-mediated soil cementation, an emerging technology which alters substantially the structure of geo-materials and endows the subsurface with improved overall mechanical behaviour. The technique integrates bacterial metabolic activity in soil-permeation solutions, to ultimately induce the formation of calcite (CaCO_3_) mineral crystals which act as binders among particles of granular soils. Quite unlike most applications targeting the artificial cementation of soils for improving their overall mechanical properties and bearing capacity^1,2^ microbial-induced calcite precipitation (MICP)^3–7^ via the ureolytic soil bacterium *Sporosarcina Pasteurii*^8^ is applied via non-erosive, low-pressure propagation of bio-chemical solutions. MICP at its core is urea hydrolysis with the catalyzing activity of the enzyme urease, which is found in several bacterial strains^9^ or other fungi and algae^9,10^. Due to the presence and activity of urease, urea hydrolysis completes 10^14^ times faster compared to the non-catalysed reaction^11^. The bio-improved geo-material can be, hence, produced *in-situ* at ambient temperature, with limited energy requirements from the relatively inexpensive combination of urea and calcium. The new, enhanced, lattice of mineral bonds among soil particles forms between several hours and few days, depending on the desired final bond content. It comprises calcite crystals which grow in number and size following hierarchical plane expansion with their dimensions ranging from few, up to hundreds of micrometers^12^. The resulting material is lightweight cemented soil which retains the permeability of its matrix^4,13^ exhibits significant enhancement of strength and stiffness^14^ and is difficult to produce by any other synthetic means.

There is still uncertainty, however, about the design and application of a controlled, large-scale soil improvement mechanism based on MICP, despite the extensive knowledge established via laboratory experimentation^15–21^. Few attempts have been made at large scale targeting real geo-technical problems^22–24^ and in some cases failed to reproduce the desired precipitation mechanism^25^. A crucial takeaway from such attempts is the improved understanding of the effect of the provided bio-chemical treatment conditions on the material’s improved mechanical response. However, the role of the intrinsic properties of the base material on the formation of the bond lattice remains relatively underexplored.

To this purpose, in the present study we adopt two different base materials and subject them to identical external treatment conditions to ultimately evaluate whether and how complex bio-chemical and transport phenomena adapt to the different available porosities. Additionally, we focus on the study of kinetics of urea hydrolysis for the case of MICP induced via utilizing lyophilized cells. The use of this cell state, which is an alternative to the direct use of vegetative cells, is considered to facilitate significantly MICP applications by overcoming the need for complex on-site bacteria cultivation^26^. It further incorporates elements of economic efficiency by lowering energy requirements and needs in resources and equipment for *in-situ* applications. Moreover, it ensures that quality control tests are carried out prior to propagating solutions in the targeted soil volume. Overall, MICP is rendered easier to reproduce, since the most crucial elements of the process, i.e. the bacterial agents, can be stored, transported and become ready to use.

Finally, although the bio-cemented geo-material was brought into focus over a decade ago^4,9^ its peculiar structure, result of bio-chemical and transport processes, has been treated solely in qualitative approaches, via surface and textural observations. In the context of the current knowledge^27–29^ microstructural information has been captured and outlined mainly through scanning electron microscopy (SEM). More precisely, the chemical composition of treatment solutions^27^, the environmental conditions^28^ and the degree of saturation^29^ during MICP improvement are all found to yield distinctive precipitation behaviors and correspond to varying obtained mechanical properties. Moreover, microstructural evidence provided in the literature is often limited to the scale of few grains and bonds and thus it remains unclear whether such evidence can be considered representative of the macro-scale.

To overcome such uncertainties regarding the limited validity of textural observations, micro-computed tomography (micro-CT) is considered herein as the ultimate tool which allows providing with a robust determination of micro-scale quantities. The goal is to capture spatial and morphological heterogeneities of micro-scale properties. Recent work in this direction has been reported in^30^ where micro-CT is mobilized for capturing microstructural quantities and their evolution with respect to changes in rheological properties, such as the porosity, permeability and effective diffusion. In the same work, comparison between the experimentally measured bond volumetric fraction and that obtained through micro-CT image processing is reported, with the mean error between the two reaching 20%. Contrary to^30^, the work presented herein deals with the micro-architecture of bio-cemented materials by implementing micro-CT to isolate and analyze individual bond particles which compose the calcite lattice in two different base materials. Results on the geometrical and spatial characteristics are interpreted and evaluated with respect to the captured strength and stiffness parameters. Moreover, complementary microstructural inspection tools are mobilized to further shed light on the bond growth mechanism and on the previously underexplored internal state of calcite bonds.

## Kinetics of cell-free MICP

Previous studies reported in the literature^5–24^ have utilized extensively whole vegetative cells of *Sporosarcina Pasteurii* which are introduced in soils to ultimately induce their bio-cementation. Herein, we obtain ureolytic cells in dry powder form through a freeze-drying process and evaluate their ureolytic capacity upon rehydration, as described in the methods section. When re-suspended in urea- and calcium-rich solutions, the lyophilized cells of *S. Pasteurii* are found to reactivate their hydrolysis mechanism and CaCO_3_ precipitates are immediately formed.

**Figure 1:**
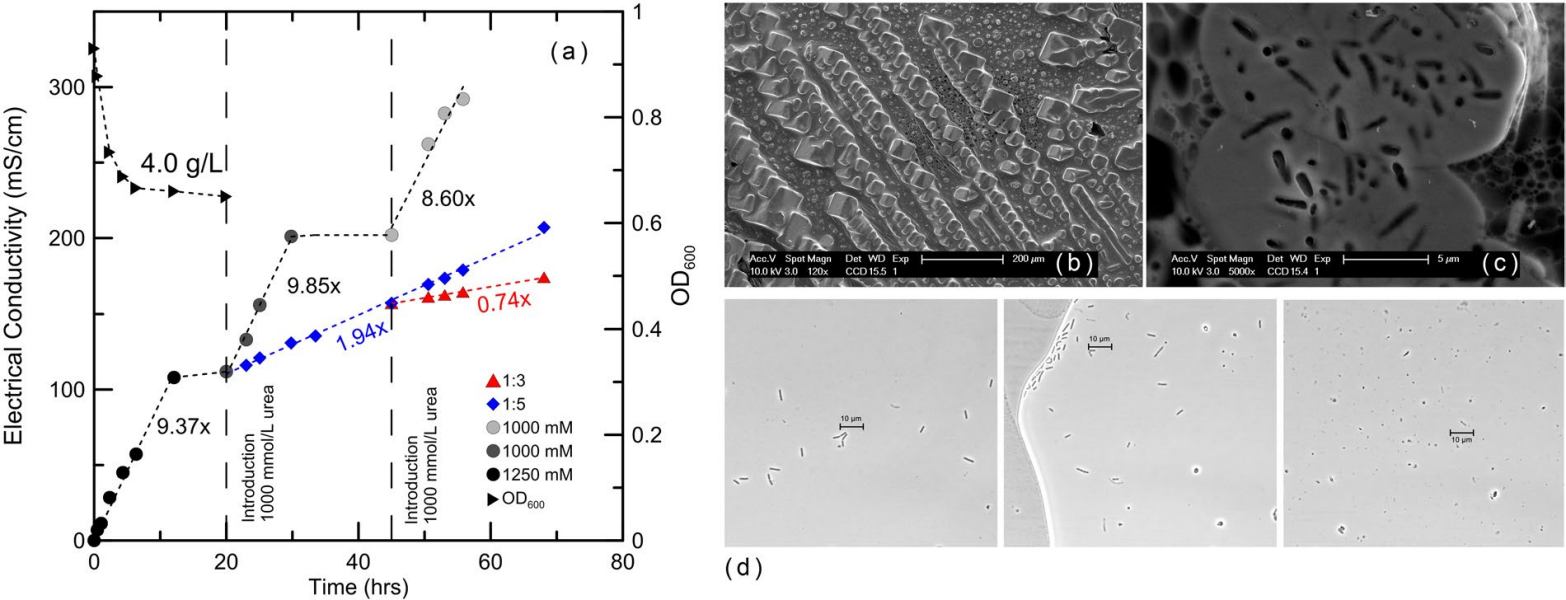
Urea hydrolysis rates (mS/cm versus time) for the initial batch which includes 4.0 g/L of rehydrated cells of S. Pasteurii. Additional dilution series (1:3) and (1:5), performed after 20 hours and 45 hours respectively, following initial resuspension of cells. Dilution refers to transferring volume of the enzyme-rich initial batch to 1 M urea, Mili-Q water batches; (**b**) CaCO_3_ cubic-shaped particles precipitated upon resuspension of the freeze-dried cells in urea- and calcium-rich solutions; (**c**) bacterial traces observed on the surface of CaCO_3_ precipitates; (**d**) (left) photomicroscopy image showing vegetative cells, taken immediately upon rehydration of the lyophilized pellet in liquid solution; (middle) image taken after 1.5 hours where dead cells start appearing; (right) image taken after 5 hours where debris and cell remnants are observed. These observations reveal a gradual breakdown of the cell clusters with remnants and debris corresponding to complete cell-wall breakdown with the corresponding optical density (OD_600_) reaching a plateau value (**a**).

Electrical conductivity serves herein as an indicator of the produced ammonium concentration, and thus of the amount of urea hydrolysed according to^9^. A maximum urea hydrolysis rate of 104 mmol/L^−1^/h^−1^ is obtained (Fig. 1a), which is within the same range as that reported in^31^ and as that found for similar biomass concentrations in^9^. These two latter studies refer to the use of vegetative cells harvested from liquid growth media and directly introduced into soils. The study of Fig. 1a reveals a continuous increase of the electrical conductivity despite complete cell lysis, which is found to occur in the first 5 hours following cell resuspension. Figure 1b reveals cubic-shaped CaCO_3_ crystals exhibiting hierarchical precipitation patterns, with traces of encapsulated cells being captured in Fig. 1c. Similar hierarchical expansion of bond particles which reproduce distinctive plane geometries has been also reported by the authors in^12^. Lysis is monitored through optical density measurements (Fig. 1a) and photomicroscopy analyses (Fig. 1d). A hypothesis based on the provided evidence is that while bacteria decay, their urease enzymes are released in the surrounding environment, carrying and executing the genetic sequences of urea hydrolysis. This “cell-free” mechanism of urea hydrolysis, captured herein, can potentially replace *in vivo* hydrolysis and facilitate real-field MICP applications by reducing installation costs and by overcoming the need of biosafety approvals according to^26^. This alternative use of freeze-dried cells is therefore expected to set the basis for the design of overall reproducible, “mainstream” soil-improvement solutions based on bio-mediated CaCO_3_ precipitation.

## Mechanics of the bio-improved geo-materials

We provide with results referring to the unconfined compressive strength (UCS) of fine- and medium-grained sand, with their identification properties listed in Table 1. It should be noted that the cumulative 50% point diameter (D_50_) of the former is almost half of that of the latter. Both materials are subjected to MICP to achieve various calcite bond contents in the range between 3–10%, by altering the number of the provided reactant batches which are rich in urea and calcium. Further details on specimen preparation are available in^12^. Same external treatment conditions are provided to sand columns where the materials are packed as a means of minimizing uncertainties related to the influence of bio-chemical and environmental conditions on the precipitated bond lattice. Thus, the morphology of the crystalline assembly of bonds, result of MICP, will be attributed only to the influence of the base materials’ initial porosity.

**Table 1:** Identification properties of the chosen sands.

	Fine	Medium
cumulative 50% point of diameter *D*_50_ (μm)	190	390
cumulative 10% point of diameter *D*_10_ (μm)	99	261
Minimum void ratio (*e*_min_)	0.56	0.69
Maximum void ratio (*e*_max_)	0.71	0.89
SiO_2_ (%)	>97	>97
Coefficient of curvature, *C*c	0.86	0.91
Uniformity coefficient, *C*u	2.10	1.6

**Figure 2:**
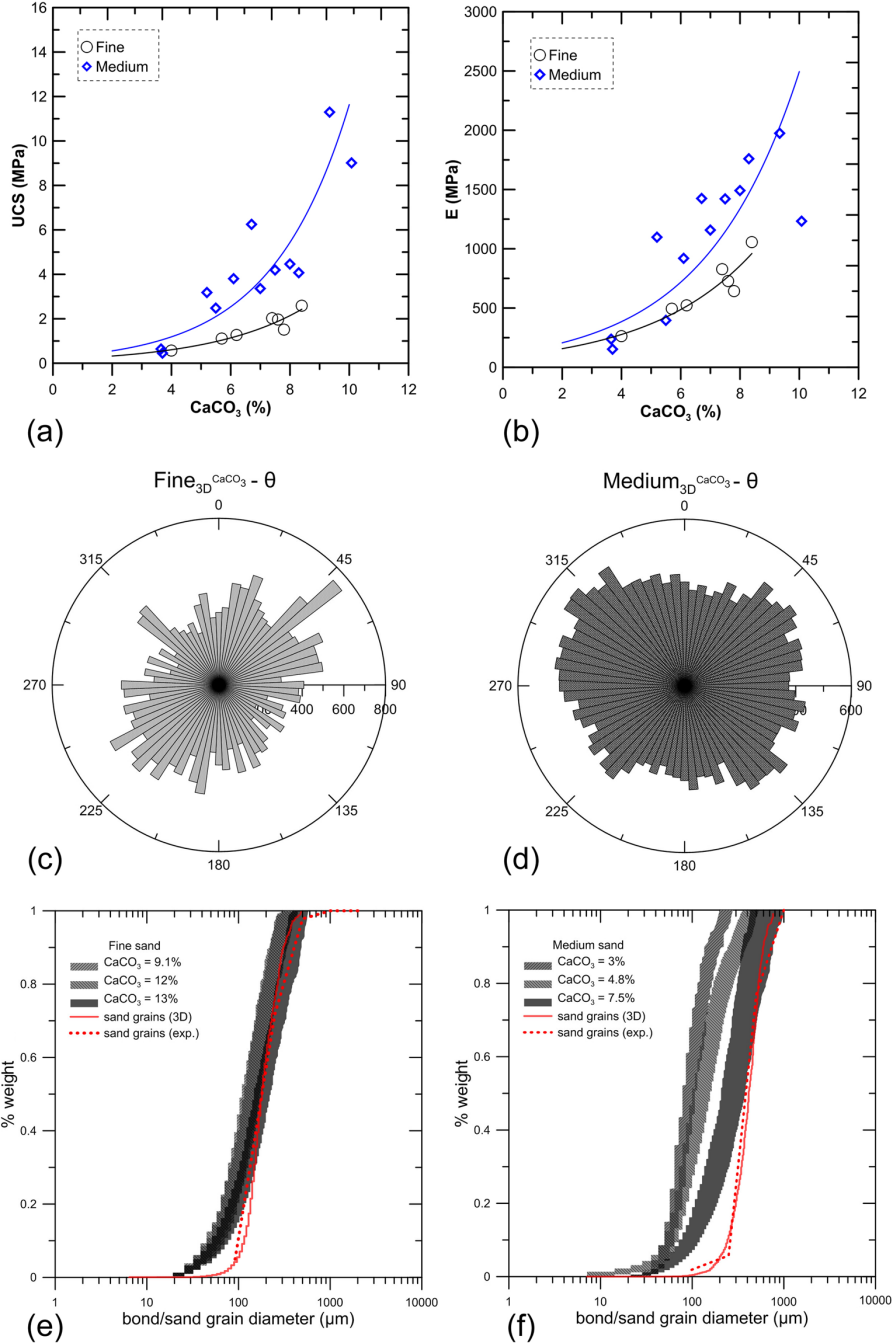
(**a**) Unconfined compressive strength for fine- and medium-grained MICP-treated specimens; (**b**) evolution of the Young’s modulus for fine- and medium-grained bio-improved sand with respect to increasing bond content; theta-orientation (θ - degrees) of the particle major axis of inertia for precipitated CaCO_3_ bonds in fine (**c**) and medium sand (**d**) of 9.1% and 7.5% bond content respectively; bond size distribution covering the range between minimum and maximum diameters of calcite bonds, presented with respect to the fraction of cumulative weight of CaCO_3_ precipitates in fine (**e**) and medium sand (**f**), for three different bond contents. Validation of the 3D reconstructed sand grains (solid) with the experimentally determined grain size distribution curve (dashed).

Interestingly, our data reveal a more pronounced strength (Fig. 2a) and stiffness (Fig. 2b) improvement for the more porous material. It should be noted that both materials yield the same peak and residual strengths in the untreated state^32^ under confinement. The UCS of medium-grained bio-improved sand falls between 3 and 12 MPa for calcite bond contents in the range of 5% to 10%. Meanwhile, the fine bio-improved sand reaches approximately 2.5 MPa for similar bond contents (Fig. 2a). The variation of the bio-improved Young’s moduli are found to exhibit similar trends with respect to increasing calcium carbonate content for both materials. For comparison, the elastic modulus of the untreated materials during unloading-reloading is estimated in the range of 200 MPa^32^. Medium-grained bio-cemented sand is found to yield higher stiffness values for all samples with their bond content greater than 5% (Fig. 2b).

The discrepancy observed in the achieved strength of bio-improved materials, which yielded similar response in their untreated state, suggests that in order to provide with a robust description of the enhanced behavior, the overall bulk mass of bonds cannot be considered as the sole factor governing the level of improvement. A better understanding of microstructural properties is hence required.

## Qualitative and quantitative microstructural characterization

**Figure 3:**
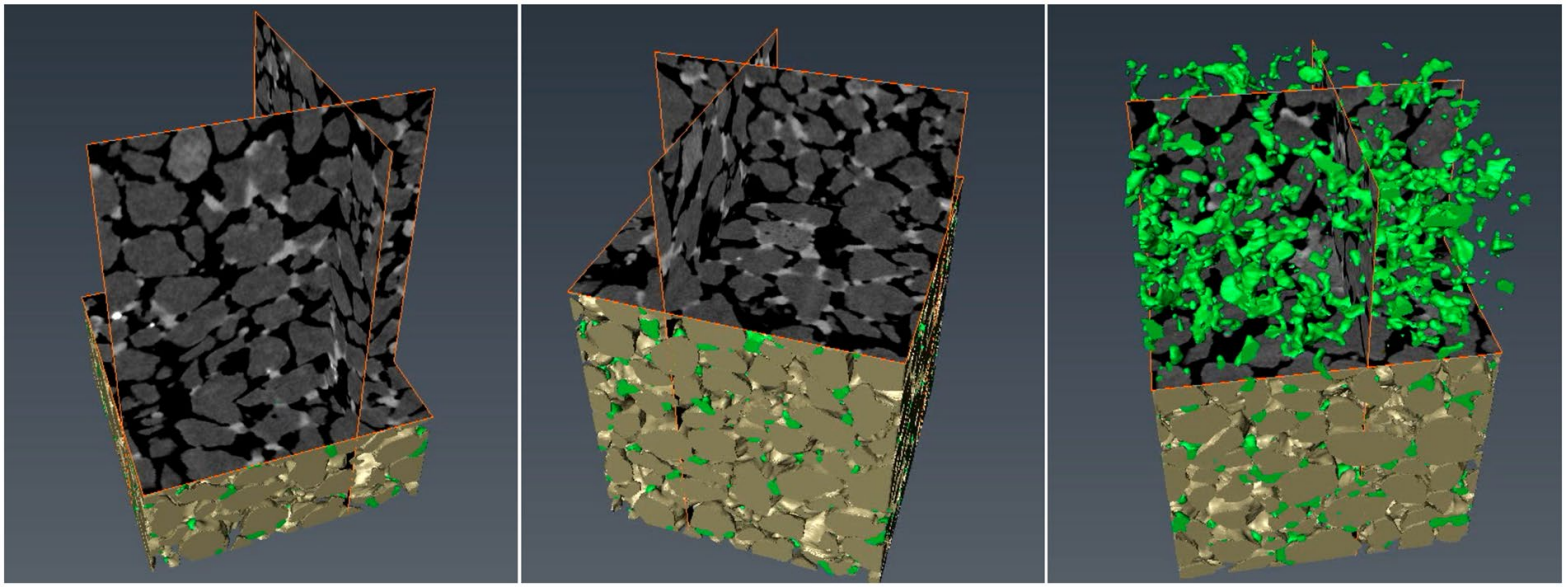
3D volume reconstruction of bio-cemented sand through combination of micro-CT scans; brown particles represent sand grains and green particles represent calcite bonds; rectangular sample with horizontal section equal to 1.5 mm^2^.

The present section comprises a description of the distinctive CaCO_3_ precipitate behaviours through 3D microstructural characterization. Figure 3 shows the process of volume reconstruction through combination of 2D X-Ray scans, where sand particles (brown) and calcite bonds (green) can be distinguished and analyzed individually. As a starting point we postulate that higher initial porosity (medium-grained sand) favors precipitation of bond particles which grow their sizes upon continuous infiltration of reactive media throughout the porous network. Furthermore, the nature of the precipitation and bond growth mechanisms is analyzed via mobilizing complementary inspection tools. Observations through qualitative inspection aim, on one hand, to serve as additional means of validation of the parameters determined via micro-CT^33^ and subsequent analysis^34^ of the reconstructed 3D solid matrix. On the other hand, alternative tools allow, for example, observing in real-time the precipitation and bond growth mechanisms, as well as better understanding the internal state of crystals.

To address the quantification of microscale parameters with respect to the calcite bond content herein is presented a methodology towards capturing the contacts between bonds and grains, as well as expressing the numbers, sizes, and particle orientations. A workflow is established and presented in the methods section referring to analyses through micro-CT. This workflow allows investigating the representary elementary volume (REV) scale to identify microstructural characteristics which are considered representative of the material’s macroscale. To this purpose, the analyzed volumes via micro-CT refer to dimensions at least ten-times the D_50_ values of the investigated materials (Table 1).

**Figure 4:**
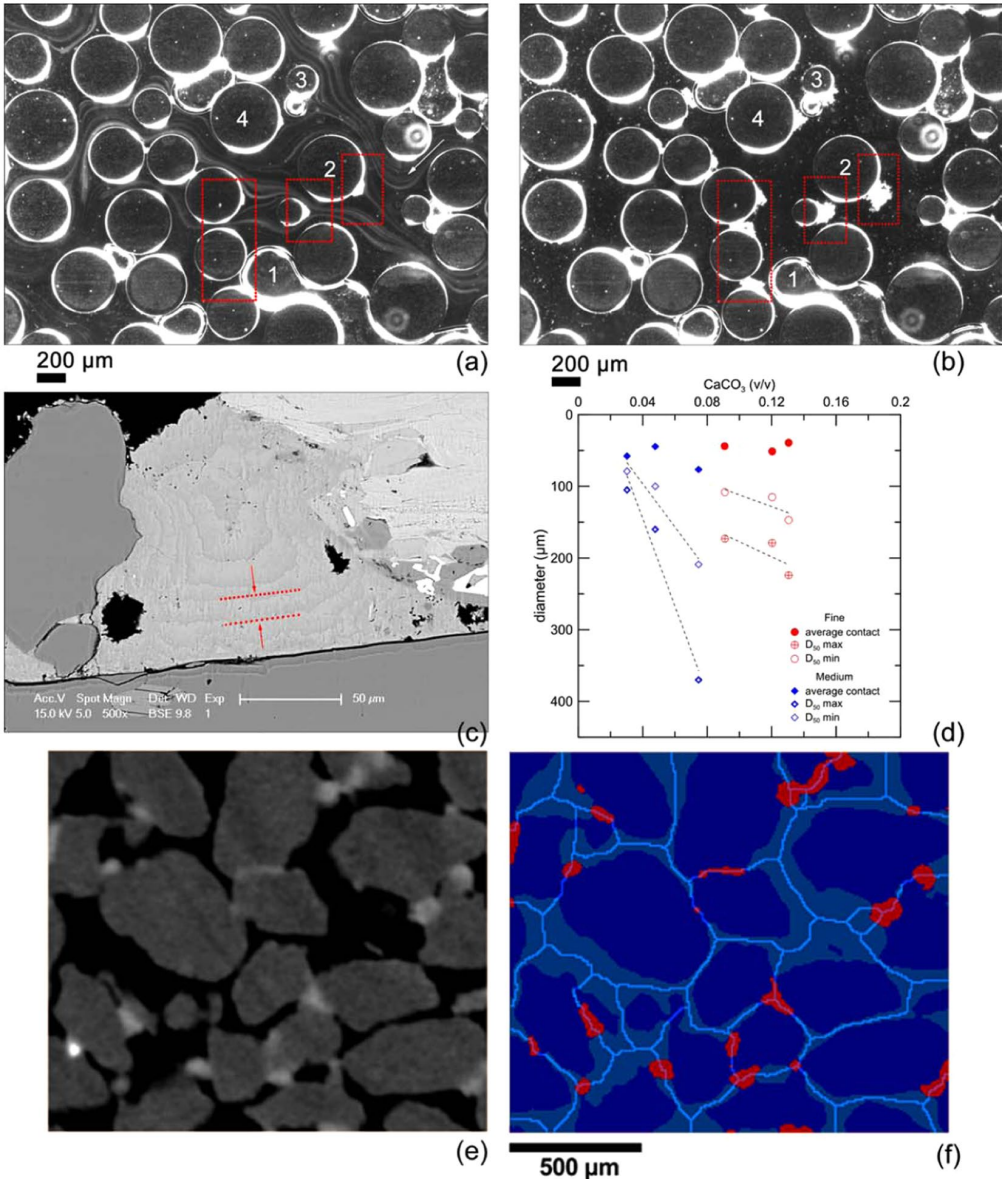
(**a**) Time-lapse video microscopy imagery taken over a period of 18 seconds (**a** to **b**) upon introduction (white arrow for the flow direction) of dissolved calcium and urea in urease-rich solution. We observe: trapped gas bubbles (no. 1), PDMS circular grains (no. 2, 3 and 4) and gradual deposition of CaCO_3_ particles (whitish particles among PDMS grains with relevant areas highlighted in red). Nuclei are transported along the flow path and accumulate on the surfaces of the model’s circular grains in areas that are less exposed to advective fluxes. For example, flow lines in the vicinity of grain No. 2 divert around it, leaving an area where crystal deposition occurs between 4a and 4b. Gas bubbles that are present in the medium, depicted in 4a and 4b, remain intact or slightly recede during the flow due to changes in the flow regime (see bubble No. 1 in a and b; (**c**) Back-scattered electron image showing the internal state of a CaCO_3_ particle that formed due to gradual deposition of individual layers with their thickness highlighted between the red dashed lines as well as traces of encapsulated bacterial cells; (**d**) summary of the evolution of mean bond diameters measured for fine- (red) and medium-grained (blue) biocemented sand, as well as the average contact diameter per bond; (**b**) raw image of bio-cemented sand obtained from micro-CT analysis; (**f**) processed image to determine the crucial contact surface between sand grains (dark blue) and calcite bonds (red) showing the split lines generated through distance transformation to determine active bonds and their diameters.

**Figure 5:**
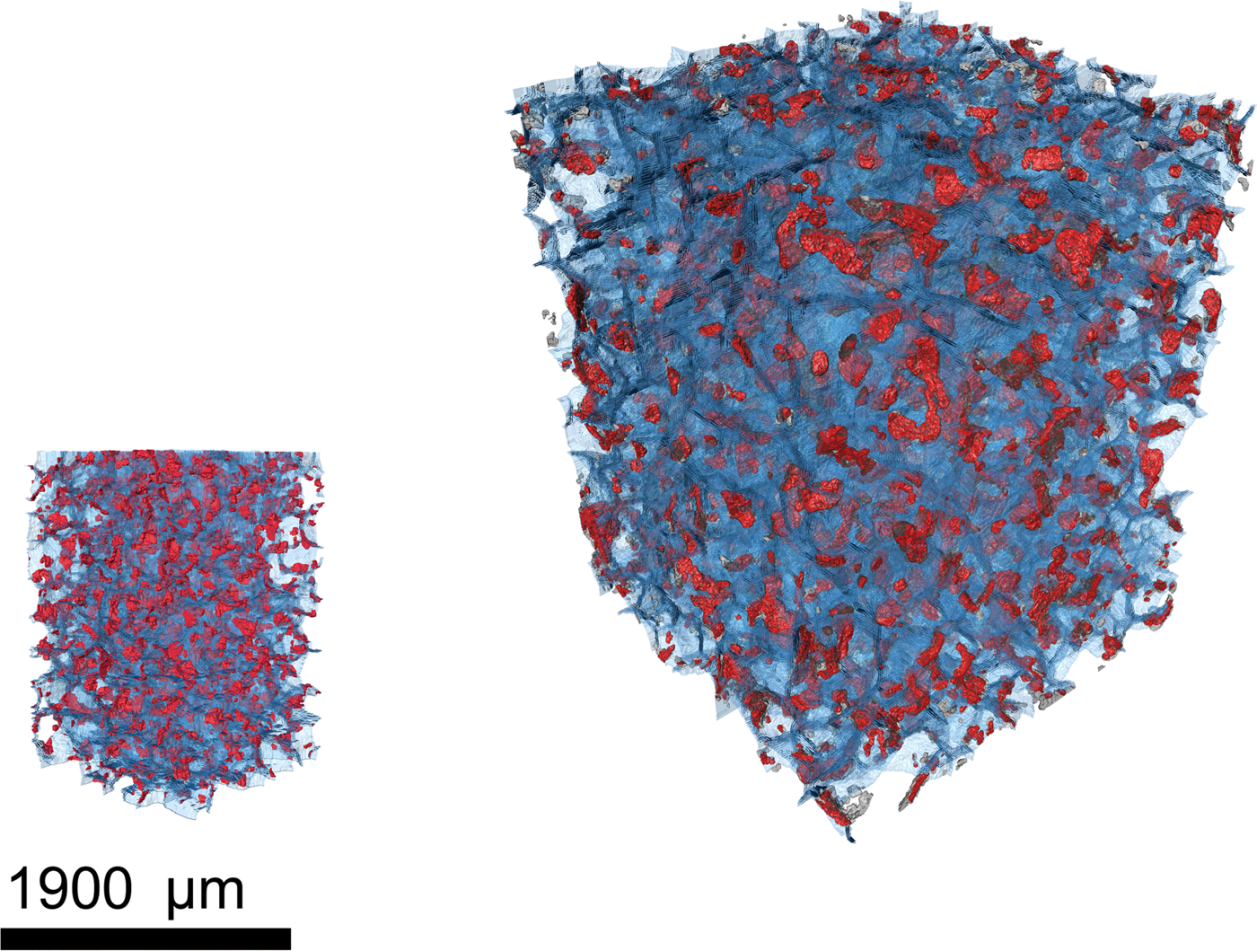
3D view of active bond areas (red) and split lines (blue) for fine- (left) and medium- (right) grained bio-cemented sand under the same scale.

The core of this digital image based approach consists in passing from a raw image obtained via micro-CT (Fig. 4e) to a segmented one (Fig. 4f) where the different solid phases, as well as the pore space, are identified. Subsequently, soil grains need to be separated since they often appear interconnected in the raw images, which hinders the accurate determination of their numbers and volumes. This separation method with distance transformation^34^ allows splitting individual soil particles. Further, by observing the intersection of bond particles with the produced split lines which divide sand particles, it becomes possible to identify the number of active bonds and the active area of contact (Fig. 4f). More precisely, bond particles which intersect with the imaginary lines splitting individual soil grains are considered as active, contributing with their planes to the transmission of normal forces and to the development of shear resistance. Active contact areas, which represent the intersection of bonds with the split lines in the three-dimensional space, are illustrated, under the same scale, in Fig. 5 for fine- and medium- grained bio-cemented sand.

**Figure 6:**
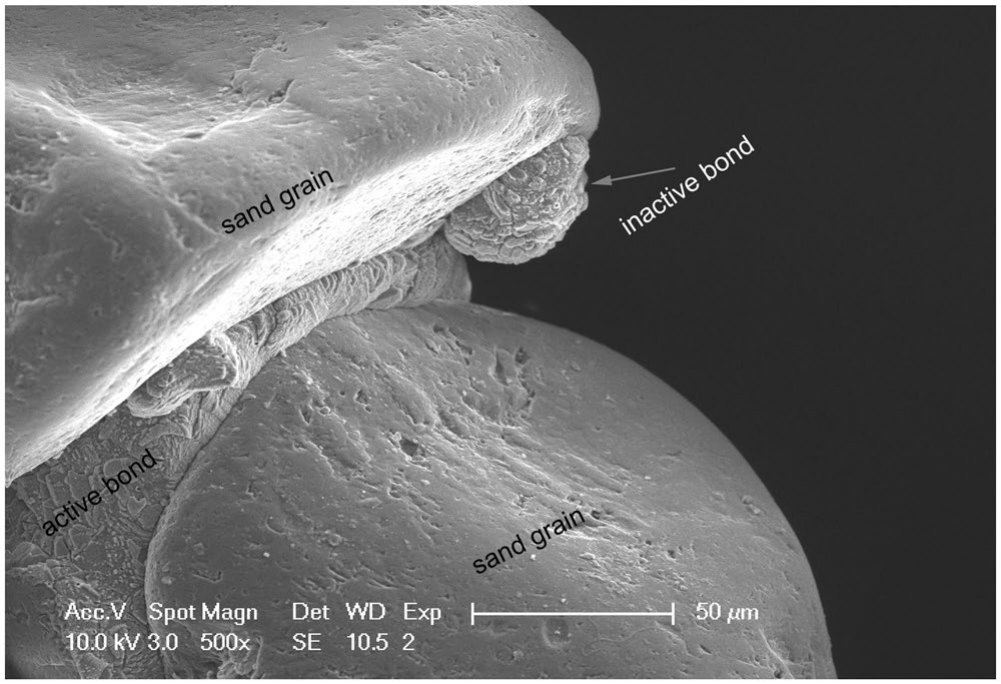
SEM image showing a real geometry of an active calcite bond which bridges two neighboring grains of sand and an inactive calcite particle.

We further suggest a bond particle analysis which expresses the particle size distribution with respect to the cumulative bond volume. This analysis follows the principle of the well-established experimental procedure for determining the grain size distribution of soils^35^. More precisely, the minimum and maximum bond diameters are used as indicators of the size range exhibited by the precipitated bond particles with respect to the cumulative fraction of the bond volume (Fig. 2e,f). In Fig. 2e and f the mean minimum (D_50_ min) and mean maximum (D_50_ max) diameters are determined as the cumulative 50% points of diameter. These values are plotted for both materials in Fig. 4d. In addition to these parameters, we define the average contact diameter (Fig. 4d) per bond as the ratio of the overall contact area between bonds and sand grains over the total number of active bonds. The average contact diameter per bond is thus a direct function of the total number of particles, contrary to the aforementioned D_50_ diameters which incorporate the notion of the cumulative volume of the bond phase. It is noteworthy to consider herein that active bonds are those which are found to bridge neighbouring soil particles. The remaining bonds are characterized as inactive since they do not contribute with their planes to inter-granular force transmission. A typical example of a real geometry and texture of active and inactive bonds is captured through scanning electron microscopy (SEM) and illustrated in Fig. 6.

The mean minimum and maximum diameters of bonds precipitated in medium-grained sand are found to range between 80–370 micrometres for calcite volumetric contents in the range between 3–7.5% (Figs 2f and 4d). Contrary, MICP treatment in fine-grained sand yields bonds with mean diameters in the range between 100–200 micrometres for calcite contents between 9–13% (Figs 2e and 4d). We postulate that larger bond particle diameters result in lower values of inter-particle stresses, in larger plane surfaces for the development of forces resisting shearing and in increased particle interlocking. This explains why increased overall resistance is captured for the medium-grained bio-cemented sand for similar bond contents with respect to fine-grained sand.

The particle orientation theta of the major inertia axis of individual bond particles is used to determine the spatial orientations of population of bonds (Fig. 2c and d). For MICP-treated medium-grained sand (Fig. 2d), bonds are found to exhibit a relatively homogenous distribution in the theta-orientation space compared to calcite bonds that precipitate in fine-grained sand (Fig. 2c). In this latter case, populations of grains yield preferential orientations around approximately 45° and 235°. We postulate that the homogeneity in the spatial distribution of crystals, and therefore of intergranular contacts along all directions, results in increased overall resistance. However, lack of particles which resist transmitting forces in a given directional range, results to weaker force chain distributions, a term commonly confronted in discrete element method (DEM) simulations^36^ and thus to lower overall shear resistance.

Results further reveal that the fraction of active bonds is estimated between 77–84% for the 6 samples analyzed through micro-CT and reconstructed numerically. The bonds per soil particle ratios are found to range between 4.3 and 3.2 for fine- and medium-grained sand respectively, independently of the overall calcite content.

We further provide a qualitative description of precipitation behaviours to understand the evolution of bond geometries with respect to the crucial overall bond content. To this purpose we perform observations via time-lapse video microscopy to obtain real-time monitoring of the evolution of MICP within a Polydimethylsiloxane (PDMS)^37,38^ physical model of a microporous medium. Observations reveal the immediate precipitation of CaCO_3_ nuclei upon the introduction of reactants and their gradual deposition on the PDMS grains’ surfaces (Fig. 4a,b). This finding suggests a growth mechanism for precipitated CaCO_3_ nuclei owing to the gradual accumulation of individual CaCO_3_ layers in areas of the porous medium that are less exposed to advective fluxes. An additional inspection tool employed herein relates to the investigation of a cross sectional area of a calcite bond through the back-scattered electron detector of a SEM microscope. The sample is previously subjected to ion-beam bombardment to ultimately obtain a planar, smoothened surface. Observation of the inner structure of calcite bonds reveals distinctive individual layers which compose a single crystalline bond (Fig. 4c). These observations validate, on one hand, the hypothesis of gradual deposition of precipitated nuclei which results to bond growth, previously observed via time-lapse video microscopy (Fig. 4a,b). On the other hand, the observed layers, as well as the traces of encapsulated bacterial cells within the CaCO_3_ bond, represent crystalline defects. The presence of defects and dislocations suggest that the engineering properties of calcite bonds in bio-improves soils, result of bio-mediated precipitation, are lower than those of pure geological calcite.

As far as the validity of the obtained results from micro-CT and image processing is concerned, we compare the geometrical characteristics determined via micro-CT and subsequent 3D volume reconstruction with parameters measured directly in the laboratory under standardized experimental procedures. More precisely we generate the 3D packing of sand particles and analyze their grain size distribution. Results, shown in solid red lines in Fig. 2e and f, are then compared against the experimentally measured curves of the particle size distribution^34^, shown in dashed red lines in Fig. 2e and f, with good agreement found between the two. Moreover, since micro-CT is a non-destructive inspection technique, the samples can be further subjected to post-scanning analyses, such as acid digestion^12^, for ultimately determining directly the crucial calcite bond content. When comparing the indirect, numerical measurements of the bond contents with direct measurements performed in the laboratory on the same samples, results reveal an error range between 2.2% and 15.1% in the total calcite content. Finally, this error between direct and indirect measurements of the calcite content confirms similar range as that reported in^30^, found to reach 20%. Such discrepancies are expected given the limitations in the maximum resolution achieved by the micro-CT equipment.

## Conclusions

The study condenses a multi-disciplinary approach to the investigation of bio-cemented geo-materials. More precisely notions of: (i) kinetics of precipitation, (ii) mechanics and (iii) microstructural inspection are brought together to provide with new insight into the contributing mechanisms and efficiency of the MICP process. The contributions of this study are summarized below based on these three principal axes.

Firstly, an alternative application of MICP for soils was introduced and discussed. Kinetics analyses showed that urea hydrolysis and calcium carbonate precipitation persist in a “cell-free” mechanism, after complete breakdown of the cell clusters of rehydrated calcifying bacteria. Additionally, the efficiency of the urease enzyme is not found to vary after application of freezing-drying and rehydration cycles or after the degradation of the cell cluster. This fundamental evidence provides with a preliminary step towards the conception of an efficient and reproducible mechanism for soil improvement based on MICP where given dry biomass yields known ranges of hydrolysis and precipitation efficiencies.

Moreover, strength and stiffness parameters are obtained for fine- and medium-grained bio-cemented samples of various overall bond contents. By applying two base materials, which yield similar response in their untreated state, to identical external treatment conditions we minimize uncertainties related to the influence of bio-chemical factors on the obtained mechanical response. Results reveal a more pronounced improvement for the medium-grained material, for similar range of the final calcite bond content. This finding confirms that the average bulk mass of bonds is not sufficient to determine the level of improvement and thus the contribution of the crystalline skeleton of bonds needs to be better understood.

In light of the captured mechanical response the study extended to cover the investigation of the three-dimensional micro-architecture of bio-cemented geo-materials. A new approach was presented which allows estimating the crucial contact area between bonds and sand grains through micro-CT analyses and subsequent 3D volume reconstruction. Microscopic parameters, such as bond sizes, numbers and orientations are determined in the representary elementary volume scale. This allows considering that the quantified parameters reflect the fabric of the material at the macroscale. Distinctive trends were obtained for the geometrical and spatial distributions of microbial-induced calcite precipitates, forming the crystalline lattice of bonds within fine- and medium-grained soils. These distinctive morphological characteristics were discussed in relation to the mechanical behaviour of the material at the macroscale.

Moreover, microscale inspection goes beyond traditional textural observations and provides with new, real-time insight into the nature of the growth mechanism and into the internal state of crystalline bonds. Deposition of precipitated nuclei was identified as a principal bond growth mechanism.

Overall, the study contributes towards an advanced characterization of the peculiar bio-cemented geo-material. A passing from qualitative approaches towards the quantification of critical parameters of its micro-architecture was presented. The improved understanding provided herein forms a new basis for the conception of modelling formulations which incorporate notions of contact mechanics or other parameters to unify microscopic quantities as a means of interpreting macroscale phenomena. Thus, the provided insight can be used to improve predictive theoretical modelling and numerical simulations. For example, a practical application of the provided insight into the peculiar architecture of the bio-improved geo-material would be to design and perform simulations using DEM^36,39–41^ on 3D particle packings where bonds represent realistic geometries and microstructural characteristics of the material. Finally, the introduced workflow to investigate, reproduce and analyse the fabric of the material can be adopted and extended to similar, natural or composite, materials with distinctive fabric properties.

## Methods

*Sporosarcina Pasteurii*^8^, which was obtained from the ATCC bacteria culture collection, is grown under sterile conditions in a liquid broth medium with its composition listed in ATCC Medium 1376. Images are collected via photomicroscopy (Nikon NI-U Microscope) at several intervals during the growth phase to evaluate the cell state and associate it with the optical density that was measured at 600 nm and ensure that no contamination occurs. An optical density equal to 1 yields 10^8^ colony-forming units (cfu) per millilitre (ml), which is determined via plating an aliquot of the liquid culture on an agar plate (with its composition mentioned in ATCC Medium 1376) using the spread plate technique.

Subsequently, the cells are centrifuged at 4 °C at 4000 g for 20 minutes. The supernatant is removed and the pellet is resuspended in liquid yeast extract medium and stored at 4 °C. Alternatively, the pellet is resuspended in the lyophilisation solution, which includes Milli-Q water, 10% NH_4_Cl and 10% sucrose. NH_4_Cl is provided at the same concentration as in the culture broth to alter the osmotic neutrality of Milli-Q water and to avoid cell lysis upon resuspension of the pellet. Sucrose is provided to maintain the integrity of the bacterial cells upon dehydration during the freeze-drying process. Upon resuspension in the lyophilisation solution, cells are stored at −80 °C for 1 hour. Subsequently, the frozen stock is placed in a vacuum chamber (Christ LCG Lyo Chamber Guard) at a minimum pressure of 0.014 bars for 24 hours. The obtained freeze-dried cultures in powder form are subsequently maintained in closed vials to protect them from atmospheric humidity and stored upon rehydration for application of MICP.

Batch experiments are carried out for measuring the electrical conductivity which corresponds to the production of ammonium and can thus be associated with the mass of urea hydrolysed. The hydrolysis of 1 mole of urea corresponds to the release of two moles of ammonium (NH_4_^+^), which results in increased electrical conductivity and alkalinity of the solution. The relationship between the total urea hydrolysed and total increase in electrical conductivity of the solution is given in^9^:

1 $${\rm{Urea}}\,({\rm{mmols}})={\rm{deltaEC}}\,({\rm{mS}}/{\rm{cm}})\times 11.11$$

As far as the microstructural analyses are concerned, we introduce a bond analysis presented in Figs 2 and 3 which uses the cumulative bond volumetric fraction as a weighting parameter. We thus follow the similar principle with the traditional determination of the grain size distribution, where the particle minimum diameters are expressed with respect to the cumulative mass fraction of population of grains^35^.

Scans were performed with the X-ray μ-CT SkyScan 1173 high energy micro-tomography scanner (Bruker MicroCT) which is equipped with a Hamamatsu 130/300X-ray beam source and a camera detector which yields images of resolutions equal to 2,240 × 2,240 pixels. Samples that are subjected to micro-CT have diameters up to 5 mm and heights up to 15 mm (micro-columns). The voxel size of the obtained images is 6.41 μm and 7.12 μm for samples of fine- and medium-grained MICP-treated sands, respectively.

A step adopted during the image processing approach shown in Figs 3, 4 and 5 lies in separating individual sand grains. As observed in Fig. 3e and f, individual contact points between soil grains are not detected via micro-CT scans due to the limitations in resolution provided by the equipment. Sand grains in the obtained raw images are connected with several pixels, hindering the estimation of their number, volume and diameters. We therefore implement a particle separation technique based on the principle of watershed lines coupled with distance transformation^34^ for ultimately obtaining the lines that split individual sand particles. An advantage of the particle separation operation performed herein is that there is no deletion of pixels, thus the particle volume remains unchanged. Upon particle separation, we perform a logical operation on the obtained binary images referring to the lines separating sand grains (namely A) and that of the segmented bond phase (namely B). For every pixel *x* which belongs to images A and B we obtain a new image C upon the logical operation described as follows:

2 $${\rm{C}}={\rm{A}}\cap B=\{{\rm{x}};{\rm{x}}\in {\rm{A}}\,{\rm{and}}\,{\rm{x}}\in {\rm{B}}\}$$

Image C, resulted from the superposition of A and B, allows determining in quantitative terms the total active contact area by analyzing the length of the split lines intersecting with bond particles in the two-dimensional space and the total area in the three-dimensional space (Fig. 5). Bonds that do not cross the split lines are considered as inactive.

### Data availability

The datasets generated and analysed during the current study related to the microstructural characterization of the materials will become available on Re3data prior to publication date. Data series obtained and analysed for the kinetics and mechanics studies are available from the corresponding author on reasonable request.

## Electronic supplementary material

Supplementary Video

## References

[1] Mihalis IK, Tsiambaos G, Anagnostopoulos A (2004). Jet grouting applications in soft rocks: the Athens Metro case. Geotechnical Eng..

[2] Vilarrasa V, Carrera J, Jurado A, Pujades E, Vázquez-Suné E (2011). A methodology for characterizing the hydraulic effectiveness of an annular low-permeability barrier. Eng Geo..

[3] Whiffin V, van Paassen L, Harkes M (2007). Microbial carbonate precipitation as a soil improvement technique. Geomicrobiol J..

[4] Mitchell JK, Santamarina JC (2005). Biological considerations in geotechnical engineering. J Geotech Geoenviron..

[5] DeJong JT (2013). Biogeochemical processes and geotechnical applications: progress, opportunities and challenges. Geotechnique..

[6] Umar M, Kassim KA, Chiet KTP (2016). Biological process of soil improvement in civil engineering: A review. J Rock Mech Geo Eng..

[7] Mujah D, Shahin MA, Cheng L (2017). State-of-the-Art Review of Biocementation by Microbially Induced Calcite Precipitation (MICP) for Soil Stabilization. Geomicrobiol J..

[8] Yoon JH (2001). Sporosarcina aquimarina sp. nov., a bacterium isolated from seawater in Korea, and transfer of Bacillus globisporus (Larkin and Stokes 1967), Bacillus psychrophilus (Nakamura 1984) and Bacillus pasteurii (Chester 1898) to the genus Sporosarcina as Sporosarcina globispora comb. nov., Sporosarcina psychrophila comb. nov. and Sporosarcina pasteurii comb. nov., and emended description of th. Int J Syst Evol Micr..

[9] Whiffin, V. Microbial CaCO3 precipitation for the production of biocement (PhD dissertation, School of Biological Sciences and Biotechnology, Murdoch University, Perth, Australia, 2004).

[10] Hamdan N, Kavazanjian E (2016). Enzyme-induced carbonate mineral precipitation for fugitive dust control. Geotechnique..

[11] Hausinger, R. P. *Biochemistry of nickel* Vol. 12 (Springer Science & Business Media, 2013).

[12] Terzis D, Bernier-Latmani R, Laloui L (2016). Fabric characteristics and mechanical response of bio-improved sand to various treatment conditions. Géotech Lett..

[13] Zamani, A. & Montoya, B. M. Permeability Reduction Due to Microbial Induced Calcite Precipitation in Sand. Paper presented at Geo-Chicago 2016: Sustainable Environmental Systems, Chicago, Illinois. Place of publication: Geotechnical Special Publication 271, Geo-Institute (August, 14–18) 2016.

[14] Harkes, M. P. *et al*. Microbial induced carbonate precipitation as ground improvement method–bacterial fixation and empirical correlation CaCO3 vs strength. Paper presented at the 1st International Conference on Bio-Geo-Civil Engineering, Delft, The Netherlands (2008).

[15] Harkes MP, Van Paassen LA, Booster JL, Whiffin VS, van Loosdrecht MC (2010). Fixation and distribution of bacterial activity in sand to induce carbonate precipitation for ground reinforcement. Ecol Eng..

[16] Van Paassen, L. A. *Biogrout, ground improvement by microbial induced carbonate precipitation* (PhD dissertation, TU Delft, Delft University of Technology, 2009).

[17] DeJong JT (2010). Bio-mediated soil improvement. Ecol Eng..

[18] Venuleo, S., Laloui, L., Terzis, D., Hueckel, T. & Hassan, M. Microbially induced calcite precipitation effect on soil thermal conductivity. *Geotech Lett*. **6**(**1**) (2016).

[19] Al Qabany A, Soga K (2013). Effect of chemical treatment used in MICP on engineering properties of cemented soils. Géotechnique..

[20] Carmona JP, Oliveira PJ, Lemos LJ (2016). Biostabilization of a Sandy Soil Using Enzymatic Calcium Carbonate Precipitation. Procedia Engineer..

[21] Cheng, L. *Innovative ground enhancement by improved microbially induced CaCO3 precipitation technology* (PhD dissertation, Murdoch University, 2012).

[22] Filet, A. E., Gadret, J. P., Loygue, M. & Borel, S. Biocalcis and its applications for the consolidation of sands. *Grouting and deep mixing*. 1767–1780 (2012).

[23] Gomez MG (2015). Field-scale bio-cementation tests to improve sands. P I Civil Eng-Ground Improvement..

[24] Van Paassen, L. A. *et al*. Scale up of BioGrout: a biological ground reinforcement method. Paper presented at the 17th international conference on soil mechanics and geotechnical engineering, Alexandria, Egypt. Lansdale IOS Press. 2328–2333 (2009).

[25] Van Paassen, L. A. Bio-mediated ground improvement: from laboratory experiment to pilot applications. Paper presented at the Geo-Frontiers 2011: Advances in Geotechnical Engineering, Dallas, Texas (March, 13–16) 2011.

[26] Volodymyr I, Chu J (2008). Applications of microorganisms to geotechnical engineering for bioclogging and biocementation of soil *in situ*. Reviews in Environmental Science and Bio/Technology..

[27] Al Qabany A, Kenichi S, Santamarina C (2011). Factors affecting efficiency of microbially induced calcite precipitation. J Geotech Geoenviron..

[28] Mortensen BM, Haber MJ, DeJong JT, Caslake LF, Nelson DC (2011). Effects of environmental factors on microbial induced calcium carbonate precipitation. J Appl Microbiol..

[29] Cheng L, Cord-Ruwisch R, Shahin MA (2013). Cementation of sand soil by microbially induced calcite precipitation at various degrees of saturation. Can Geotech J..

[30] Dadda A (2017). Characterization of microstructural and physical properties changes in biocemented sand using 3D X-ray microtomography. Acta Geotech.

[31] Lauchnor EG (2015). Whole cell kinetics of ureolysis by Sporosarcina pasteurii. J Appl Microbiol..

[32] Terzis, D. & Laloui, L. *On the Application of Microbially Induced Calcite Precipitation forSoils: A Multiscale Study* (eds Ferrari, A. & Laloui, L. Springer Series in Geomechanics and Geoengineering, Springer, Cham 2017).

[33] Ketcham RA, Carlson WD (2001). Acquisition, optimization and interpretation of X-ray computed tomographic imagery: applications to the geosciences. Comput Geosci..

[34] Avizo user’s guide. FEI company – Thermoscientific (2009).

[35] ASTM D6913/D6913M-17. Standard Test Methods for Particle-Size Distribution (Gradation) of Soils Using Sieve Analysis. ASTM International, West Conshohocken PA. (2017).

[36] Wang YH, Leung SC (2008). A particulate-scale investigation of cemented sand behavior. Can Geotech J..

[37] Yang W (2010). Fabrication of a Hydrophilic Poly (dimethylsiloxane) Microporous Structure and Its Application to Portable Microfluidic Pump. Jpn J Appl Phys..

[38] Jiménez-Martínez J (2015). Pore-scale mechanisms for the enhancement of mixing in unsaturated porous media and implications for chemical reactions. Geophys Res Lett..

[39] Evans, T. M., Khoubani, A. & Montoya, B. M. Simulating mechanical response in bio cemented sands. Paper presented at the 14th international conference of the international association for computer methods and recent advances in geomechanics: *IACMAG* 2014, Kyoto, Japan (Taylor and Francis, Balkema, 2015).

[40] Feng K, Montoya BM, Evans TM (2017). Discrete element method simulations of bio-cemented sands. Comput Geotech..

[41] Yang P, O’Donnell S, Hamdan N, Kavazanjian E, Neithalath N (2017). 3D DEM Simulations of Drained Triaxial Compression of Sand Strengthened Using Microbially Induced Carbonate Precipitation. Int J Geomech..

